# MEvoLib v1.0: the first molecular evolution library for Python

**DOI:** 10.1186/s12859-016-1303-3

**Published:** 2016-10-28

**Authors:** Jorge Álvarez-Jarreta, Eduardo Ruiz-Pesini

**Affiliations:** 1 Depto. de Informática e Ingeniería de Sistemas (DIIS), Universidad de Zaragoza, María de Luna 1, Zaragoza, 50018 Spain; 2 Instituto de Investigación en Ingeniería de Aragón (I3A), Universidad de Zaragoza, Mariano Esquillor s/n, Zaragoza, 50018 Spain; 3 Depto. de Bioquímica, Biología Molecular y Celular, Universidad de Zaragoza, Miguel Server 177, Zaragoza, 50013 Spain; 4 Instituto de Investigación Sanitaria de Aragón (IIS Aragón), San Juan Bosco 13, Zaragoza, 50009 Spain; 5 CIBER de enfermedades raras, Instituto de Salud Carlos III, Monforte de Lemos 5, Madrid, 28029 Spain; 6 Fundación ARAID, María de Luna 11, Zaragoza, 50018 Spain

**Keywords:** Molecular evolution, Multiple sequence alignment, Phylogenetic inference, Supertrees, Biological knowledge, Software, Python

## Abstract

**Background:**

Molecular evolution studies involve many different hard computational problems solved, in most cases, with heuristic algorithms that provide a nearly optimal solution. Hence, diverse software tools exist for the different stages involved in a molecular evolution workflow.

**Results:**

We present MEvoLib, the first molecular evolution library for Python, providing a framework to work with different tools and methods involved in the common tasks of molecular evolution workflows. In contrast with already existing bioinformatics libraries, MEvoLib is focused on the stages involved in molecular evolution studies, enclosing the set of tools with a common purpose in a single high-level interface with fast access to their frequent parameterizations. The gene clustering from partial or complete sequences has been improved with a new method that integrates accessible external information (e.g. GenBank’s features data). Moreover, MEvoLib adjusts the fetching process from NCBI databases to optimize the download bandwidth usage. In addition, it has been implemented using parallelization techniques to cope with even large-case scenarios.

**Conclusions:**

MEvoLib is the first library for Python designed to facilitate molecular evolution researches both for expert and novel users. Its unique interface for each common task comprises several tools with their most used parameterizations. It has also included a method to take advantage of biological knowledge to improve the gene partition of sequence datasets. Additionally, its implementation incorporates parallelization techniques to enhance computational costs when handling very large input datasets.

## Background

Molecular evolution studies have always involved hard computing problems [1, 2], even more since the development of the *next generation sequencing* [3]. This has increased almost exponentially the amount of available biological information, moving molecular evolution studies into the Big Data category. However, there is one main drawback: some methods have become obsolete due to their utmost time cost to process large amounts of data.

Molecular evolution workflows usually involve a multialignment process, for which an optimal solution has a computational complexity of *O*(*m* ^*n*^), where *m* is the length of the sequences and *n* is the total number of sequences to align. This has led to the creation of many heuristic tools and methods to perform good approximations to the optimal multialignment. Moreover, the ideal phylogenetic tree construction requires the computation of the best topology for the input data, where, for *k* leaves, the number of possible binary unrooted topologies is equal to (2*k*−5)!/[2^(*k*−3)^(*k*−3)!] [4]. Thus, another group of heuristic algorithms has been developed in order to achieve a nearly optimal phylogenetic inference procedure.

Therefore, molecular evolution studies require to determine the tool and parameterization that is most appropriate for the stated requirements and the data to be processed. Hence, some software libraries like Biopython [5] have been created to allow users to work with different bioinformatics tools in an easy way through different programming languages. However, these libraries are often of general purpose, neglecting many different existing tools for specific fields and tasks, like the phylogenetic inference process. Furthermore, these interfaces do not always grow with the parameterization extension of their related tools, making them suitable only for specific versions.

In this paper we present MEvoLib, the first molecular evolution library for Python. It provides a unique interface for each task involved in common molecular evolution studies, including several tools and methods per task. MEvoLib is freely available and it has been implemented to handle even large-case scenarios. It also includes a novel method that takes advantage of available biological knowledge to improve the gene extraction of an input dataset of sequences.

## Implementation

**Fig. 1:**
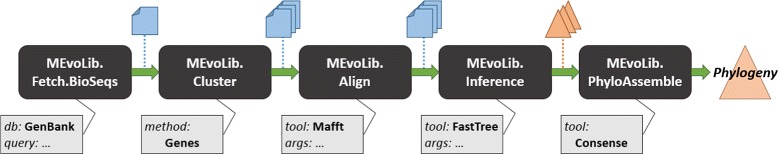
Motivating example of the application of MEvoLib. The figure shows a common molecular evolution workflow: data fetching, clustering, multialignment, phylogenetic inference and building of the consensus tree. Each stage presents the MEvoLib’s interface for that procedure and an example of a possible parameterization. The sequence sets and subsets are represented by sheets and the resultant phylogenies by triangles

MEvoLib covers the most important tasks involved in molecular evolution studies, including data fetching and clustering, multiple sequence alignment, phylogenetic inference and phylogenetic assembly (Table 1). This last procedure covers both consensus tree and supertree estimations. Furthermore, some clustering methods take advantage of the available biological knowledge to improve their accuracy. Figure 1 shows a motivating example of the usage of MEvoLib. It displays a usual phylogenetic inference workflow that will be further explained in the next section with a real case study of two specific genes of the hmtDNA (*human mitochondrial DNA*).

**Table 1:** MEvoLib modules

Category	Module list
Data	rCRS
Fetch	BioSeqs, PhyTrees
Cluster	Naïve rows, Naïve columns,
	PRD, Genes
Align	Mafft, Clustal Omega,
	Muscle
Inference	FastTree, RAxML
PhyloAssemble	Consense

### Data fetching

MEvoLib provides two different classes to fetch biological information, either from local files or from different NCBI databases. The *BioSeqs* class can fetch biological sequences from both sources, whilst the *PhyTrees* class fetches phylogenetic trees only from local files. Both classes can work with any file format supported by Biopython. Once all the desired information is gathered, it can be written in a GENBANK or NEWICK file format, respectively, along with a report file (saved with *.rep* extension and the same filename). This last file encloses the number of sequences or trees stored, together with a detailed list of all the data sources, which includes the date and time when the source was accessed and its origin, i.e. complete path of the local file or the NCBI database and query.

The report file strengthens the experiment reproducibility, not only storing all the sources’ information, but also with a temporal line that keeps track of any data overriding due to an identifier collision from two or more sources. Moreover, the report file can replace the data file when sharing or publishing research files, especially when the data file is large and the source is not a local file.

*BioSeqs* implements three useful properties to fetch sequences from NCBI databases (e.g. GenBank [6]). The first one consists of a size limitation on the number of sequences fetched for the given database and query, intended for particular researches where the user might just want to retrieve a representative sample. The second feature enhances the download process of large datasets, computing the adequate batch size for the expected sequence’s size (in bytes). This calculation is based on the directions given by the NCBI itself, together with a prefixed maximum download time for each batch to optimize the interval and number of requests made. This upper bound also reduces the data loss when an unexpected communication error happens. The third property enhances the update process, removing those sequences that are no longer stored in the corresponding NCBI database, and fetching only those sequences that are new or updated (based on their accession’s version).

### Embedding biological information

A few methods included in MEvoLib take advantage of biological knowledge to improve their accuracy. Therefore, the library includes certain reference sequences and their related information (e.g. gene loci), as well as some base phylogenies (e.g. the hmtDNA’s haplogroup phylogeny). Hence, we avoid unnecessary internet connections to retrieve the data at runtime, preventing potential errors and hastening the methods.

### Data clustering

The *Cluster* interface has been designed to provide four different approaches to split an input sequence dataset into smaller subsets. Those four methods are: naïve rows, naïve columns, PRD (*padded-Recursive-DCM3 decomposition* from DACTAL [7]) and genes. Many of them include parallelization techniques to improve their performance, especially in large-scale scenarios. Each method returns the clustering as a dictionary, with set identifiers as keys and one list of *SeqRecord* objects (from Biopython) per key as values.

The *naïve rows* method divides the input sequences into the given number of disjoint sets, with each set containing roughly the same number of sequences. The *naïve columns* method calculates the fragment length (and the range indices) based on the longest input sequence and the given number of sets.

The *PRD* method clusters the input sequences into subsets of complete sequences with the specified amount of overlapping. The user must provide an input phylogeny for the given sequences, a maximum set size and an overlapping value. The software tool uses a phylogenetic-driven methodology to obtain the overlapping subsets. The method is applied recursively to those sets that exceed the maximum size threshold, extracting their corresponding subtree from the original phylogeny. This method has been improved by switching the recursive part to a parallelized iterative procedure, where a queue manages the sets which require further decomposition.

The last method (*Genes*) has been designed to generate as many sets as genes available in the input sequences. The main idea is to take advantage of the biological knowledge to extract all the genes of a set of sequences. We have created a small database with all the available features and their qualifiers at NCBI databases. Thus, users can append a feature filter to extract only those ones in which they are interested. The *Genes* method uses the biological information available in the input file (GENBANK format) to determine the gene loci of each sequence. We have also included the method we published for PhyloFlow [8, 9] to handle information-less sequences. It uses the corresponding RefSeq (*reference sequence*) for the input dataset (indicated by the user) to generate a pairwise alignment of the information-less sequence with the RefSeq. After that, it removes the sites corresponding to new gaps introduced in the RefSeq and, finally, it applies the gene locus information of the RefSeq to the new sequence. We wanted to extend the usage of this method to expose possible errors in the information available: NCBI databases, like GenBank, do not perform any standardization over the values of each qualifier and feature of each new sequence, generating several nomenclatures for the same gene. Thus, the *Genes* method implements a statistical sampling equation to detect those related terms for the same gene with a low sampling representation. As a result, a log file is created with each uncertain pair of terms and a list with the sequences that support them. Hence, the user can easily detect sequences which merge two genes due to an error in their information. A detailed example is given in the “Results and discussion” section.

### Multiple sequence alignment

Many phylogenetic inference tools demand a MSA (*Multiple Sequence Alignment*) as input to be able to apply their heuristics to build the phylogeny. Therefore, our library includes an *Align* interface to work with different MSA tools. The main characteristic of this interface is that there is only one parameterization for every MSA tool included in MEvoLib. The user has to indicate the tool to launch, the input sequence file, the file format and the list of arguments of the tool. The resultant alignment will be returned in a *MultipleSeqAlignment* Biopython object. Also, an output file (and its format) can be added to the previous list of parameters in order to save the resultant MSA. The purpose of the input and output file formats is twofold: **i)** to ensure that we can read and write in those formats (through Biopython); and **ii)** to apply format conversion if the input or output formats are not supported by the MSA tool. The latter facilitates the work of the user and it improves the interface’s usefulness in scenarios where the next process requires a specific file format.

For each MSA tool included in MEvoLib, we have created a configuration dictionary, where common parameterizations are referenced by a keyword that can be passed to the *Align* interface instead of the list of arguments. This addition improves the readability of the code and should also facilitate recalling the whole set of configurations. The user can also include other keywords and configurations in the corresponding MSA tool file of the library. We have included in every dictionary a *default* keyword, which will be used if no configuration parameters are provided. It is also expected to ease the initial work of novel researchers.

In this first version of MEvoLib, we have included three of the most used MSA tools: Mafft [10], Clustal Omega [11] and MUSCLE [12]. Moreover, the method can launch MSA tools not included in the installed version of the library. To do so, the user must include two new elements into the previous parameter list: a list of supported input file formats and the input file command (e.g. *–in*). However, this functionality is intended for expert users only.

### Phylogenetic inference

The *Inference* interface handles the phylogenetic inference process with widely used software tools. As with *Align*, this interface has a single parameterization for every inference tool included in MEvoLib. The user must provide the desired phylogenetic estimation tool, the input sequence file and its format, the list of arguments of the tool and the number of bootstraps to generate during the inference process (only available for those tools that include this functionality). The resultant phylogeny will be returned in a *Tree* object, from Biopython, together with its log-likelihood score. The list of parameters can be extended to include the output tree file and its format, where the phylogenetic tree will be saved. *Inference* also includes the aforementioned automatic format conversion functionality, which facilitates the interaction of MEvoLib with other methods and software tools.

This interface also implements a dictionary of common configurations for each included tool. Apart from the *default* configuration, which is used when no list of arguments is presented, all the keywords follow the same nomenclature: [*evolution model*]+[*approximation method*]. It has been designed to resemble the model applied with the predefined configuration, improving both the code readability and the keyword memorization. New configurations included by the user may ignore this nomenclature.

The presented version of MEvoLib supports two phylogenetic inference tools: FastTree [13] and RAxML [14].

### Phylogenetic assembly

Although the *PhyloAssemble* interface is intended for consensus tree and supertree estimation processes, in the current version of MEvoLib only the consensus tree estimation process has been implemented. To get the consensus tree from a set of phylogenetic trees, the interface requires the consensus tree tool, the input tree file, the file format, and the list of arguments of the tool. The resultant consensus tree will be returned in a *Tree* object, from Biopython. It can also be saved as a tree file including in the parameter list its file path and format. *PhyloAssemble* also includes the automatic format conversion functionality.

This first version of MEvoLib incorporates a modified version of the Consense method from PHYLIP [15] for the consensus tree estimation process. The source code has been changed to allow two new arguments: the input and output file paths. The reason for such a decision is that the default behavior of Consense is to read a file named *infile* in the current working directory and to write the consensus tree in a file named *outfile* in the same directory; this may lead to execution problems in infrastructures like clusters, e.g. the working directory in the node running the job might not be writable by the user, raising an error in runtime.

## Results and discussion

### Test suite

The first version of MEvoLib includes a test suite that can be launched before installing the library to check if each module will work properly. We have arranged 7 small datasets to carry out the tests, composed by biological sequences from public datasets. The source information is available in the README file within the *Test* folder of the library.

The test suite will evaluate each functionality provided by each module of the library, displaying any error that may raise during the tests. Those modules that require any external software, like the MSA module, will only test their functionality if the expected software tools have been installed in their default installation paths. Otherwise, a warning message will be displayed, but it will not be taken as a test error.

### *Genes* method performance

The *Genes* method uses only the biological knowledge provided by the user in the sequences file, preventing any guided or prior information in the source code (apart from misspelling checkers). Thus, our method is very likely to run accurately even if the information changes or the classification criteria are modified due to new discoveries. The method collects all the available information of each sequence and it determines which parts of a set of sequences refer to the same gene using algebra of sets. This methodology works properly even though the genes might be in different loci.

The classical approach to withdraw different genes from a set of sequences only extracts information from *gene* or *product* qualifiers from the features provided by GenBank. To demonstrate the improvement that the *Genes* method offers in comparison to the classical approach we have downloaded all the complete hmtDNA sequences available at GenBank (on 07/Jul/2016). The 31755 sequences match the following query: *“homo sapiens”[porgn] AND mitochondrion[Filter] NOT mRNA[Filter] AND “complete genome”*. We have also created three subsets with the first 100, 1000 and 10000 sequences of the downloaded data to perform an additional scalability test for our method.

Before running all the tests, we have executed the *Genes* method with all the sequences in its default configuration to analyze the log file to detect possible errors in the biological information. Three sequences have been removed from the downloaded set: **i)** KP702293.1 has the gene ND3’s product as “NADH dehydrogenase subunit 2”; **ii)** FR695060.1 has the CDS “ATP synthase 6” identified as ATP8; and, **iii)** DQ862537.1 has the gene ATP6’s product as “cytochrome c oxidase subunit III”. The three subsets have also been regenerated.

To perform the classical approach, we have modified the default behavior of the *Genes* method: instead of collecting the information of all the qualifiers of each feature, it only took into account the *gene* or *product* qualifiers, respectively. All the results can be found in Table 2. We have focused our conclusions on the recovery rate of the CDS (*coding DNA sequence*), rRNA (*ribosomal RNA*) and tRNA (*transfer RNA*) features for each configuration. The hmtDNA contains 13 CDS, 2 rRNA and 22 tRNA genes.

**Table 2:** Performance and feature recovery of a classical approach versus *Genes* method of MEvoLib for four sets of hmtDNA sequences

Num. Seqs.	Configurations	Time (s)	Mem. (MB)	CDS feat.		rRNA feat.		tRNA feat.	
100	Gene	1.56	32.37	26	(+13)	3	(+1)	21	(-1)
100	Product	1.41	32.33	18	(+5)	2	(0)	20	(-2)
100	All	1.42	32.46	15	(+2)	2	(0)	20	(-2)
1000	Gene	13.15	91.42	26	(+13)	5	(+3)	43	(+21)
1000	Product	12.71	92.06	22	(+9)	4	(+2)	20	(-2)
1000	All	13.74	92.63	15	(+2)	2	(0)	20	(-2)
10000	Gene	127.95	687.63	31	(+18)	5	(+3)	45	(+23)
10000	Product	171.80	692.59	34	(+21)	4	(+2)	20	(-2)
10000	All	136.91	700.73	13	(0)	2	(0)	20	(-2)
31752	Gene	412.22	2126.83	33	(+20)	5	(+3)	46	(+24)
31752	Product	467.78	2144.32	51	(+38)	6	(+4)	20	(-2)
31752	All	509.78	2177.28	13	(0)	2	(0)	20	(-2)

The recovered features are CDS, rRNA and tRNA. The classical approach uses the qualifiers *gene* or *product* separately, whilst the *Genes* method exploits *all* of them. The second column of each feature shows the result’s divergence from the expected value

Table 2 shows that the time and memory usage of the *Genes* method is slightly higher than classical approaches in most cases, but the difference is negligible in comparison to the improvement in the recovery rates. The rRNA genes are always clustered accurately, even when the number of sequences is increased. The CDS genes are correctly gathered when the number of sequences is high enough. For instance, for the 100 dataset, the variety of information available is not large enough and, thus, the *Genes* method obtains 15 genes instead of 13 because it finds two nomenclatures for 2 different genes: “atp6” and “atpase 6”, and “atp8” and “atpase 8”. The tRNA feature is never recovered completely due to a duplication of two tRNAs in the hmtDNA^1^.

### Biological example: *MT-ATP6 and MT-ATP8 genes*

In this section we present an example illustrating the potential of MEvoLib following the workflow presented in Fig. 1. Our study is focused on the molecular evolution of two hmtDNA genes: *MT-ATP6* and *MT-ATP8*. We want to know if they have evolved similarly based on the following information: **i)** both genes encode proteins that belong to the same subunit of the ATP synthase enzyme; and, **ii)** these two genes are located in the same strand and they have an overlapping of 42 bp.

First, we are going to download all the hmtDNA sequences from GenBank that have any information related with the *MT-ATP6* or *MT-ATP8* genes. The basic query to achieve this purpose would be: *“homo sapiens”[porgn] AND mitochondrion[Filter] NOT mRNA[Filter] AND (atp6 OR atp8)*. But based on the information we have gotten in our study of the *Genes* method, these genes are referred with different nomenclatures. Therefore, our fetching source code is:



We would download 32548 sequences with the first query, whilst the second one fetches 32626 sequences (on 07/Jul/2016). We have aforementioned three hmtDNA sequences that have errors in their biological information, puzzling the genes’ extraction. Hence, we are going to remove from the downloaded dataset only those sequences that interfere with our study, i.e. DQ862537.1 and FR695060.1:



Next, we need to extract the genes we are interested in from the filtered dataset. For this example, we are going to focus exclusively on the CDS feature. In a complete study, we would also require the *gene* feature to include those sequences that might have only tagged the genes under this feature. The following source code presents the gene extraction applying the *Genes* method:



Afterwards, we extract those elements in the dictionary that refer to the *MT-ATP6* and *MT-ATP8* genes and we save the corresponding sequence fragments in two separated FASTA files. The software tool we are going to use to build the phylogenetic trees expects an alignment as input. Thus, the following code implements the multialignment procedure for the *MT-ATP6* gene using Mafft in its default configuration (*--auto*):



The next stage in our workflow is the phylogenetic inference. We are going to use the *Inference* interface from MEvoLib, selecting the FastTree tool in its default configuration (*GTR+CAT evolution model*). The resultant *MT-ATP6* gene tree and its maximum likelihood score will be returned, and the tree will also be saved in a NEWICK file:



The inference workflow for this research would be completed with these last results. To extend our study to include the last stage of the workflow shown in Fig. 1, we could be interested in analyzing the consensus tree composed from the *MT-ATP6* phylogenies of different species, using the Consense method from PHYLIP in its default configuration (*majority rule consensus*):



In the current version of MEvoLib, the further analysis of the resultant phylogenies requires supplementary software tools.

### MEvoLib and Biopython

MEvoLib takes advantage of specific classes and modules of Biopython. This design decision has been made in order to provide integrity with other tools and scripts that use Biopython, as well as to avoid the replication of already functional and public modules.

The creation of MEvoLib was encouraged by two important shortcomings found in Biopython. The first one is the lack of flexibility with the supported software tools. For instance, the parameter list of the Mafft module is fixed and not completely related with the arguments the software tool might accept, and thus the user must learn and comprehend both interfaces in order to make Mafft run through Biopython. Furthermore, the parameter list has not changed along with the new versions of the software, so the new functionalities (e.g. *--add* feature introduced in version 7.273) are not supported.

The second shortcoming is its scope. Biopython has been designed as a biological computation library. Therefore, it is incomplete for molecular evolution researches, missing support for important software tools and methods required in this specific field. MEvoLib meets these requirements, establishing a symbiosis with Biopython.

### Future development

We plan to include more software tools for the different interfaces already included in the presented version of MEvoLib. For instance, MEvoLib will include PRANK [16] as a new MSA tool, a haplogroup clustering for sequence types like the hmtDNA, PhyML [17] for phylogenetic inference, a bootstrapping method (Seqboot of PHYLIP [15]) to apply when the inference method cannot generate them by itself, and SuperFine [18] as an example of supertree construction methods.

We propose to change the design of the fetch methods from a complete memory storage of all the biological information to a small memory indexation of a file or files in the hard drive. This rearrangement of storage would slightly slow down the method’s performance but it would remarkably increase the amount of manageable data. Moreover, we aim to parallelize methods like *Genes* to reduce their time cost for very large scenarios. We also intend to include phylogenetic analysis methods, e.g. the conservation index method we presented in 2015 [19].

Finally, we contemplate to improve the test suite with an option to introduce the path of the required external software tools when they are not found in their default one.

## Conclusion

MEvoLib, the first molecular evolution library freely available for Python, is presented in this paper. MEvoLib offers a wide range of modules and methods that cover all the main stages involved in usual molecular evolution workflows. The fetching method with an historical track of the sources enhances the experiment reproducibility. Our library has been designed to provide full integration with many widely used molecular evolution tools, such as Mafft or FastTree, besides a complete interface that facilitates the work of both expert users and novel ones. The automatic format conversion facilitates the transition between stages in the workflow. Moreover, we have demonstrated that MEvoLib includes methods aimed to take advantage of the currently available biological knowledge to significantly improve on existing techniques. Finally, MEvoLib has been designed and implemented to improve the performance of existing methods and software tools for large-case scenarios. We have also discussed the improvement that MEvoLib offers to phylogenetic studies in comparison with other existing bioinformatics libraries for Python, like Biopython.

We include a manual in the software repository to provide a better understanding of MEvoLib capabilities and how to use it. Besides, the manual covers two step-by-step examples of biological studies performed with this library.

## Availability and requirements

- **Project name:** MEvoLib
- **Project home page:** http://github.com/JAlvarezJarreta/MEvoLib
- **Archived version:** https://zenodo.org/record/58486
- **Operating system(s):** Linux, Windows and MacOS
- **Programming language:** Python
- **Other requirements:** Python 2.7 or Python 3.3, NumPy 1.10.1, Biopython 1.67 and DendroPy 4.0.3 (or newer versions of all of them).
- **License:** GNU GPL
- **Any restrictions to use by non-academics:** None

The datasets supporting the conclusions of this article are available at the ZARAMIT group site, http://zaramit.org/datasets.

## Endnote

^1^ The tRNAs for Leucine and Serine have two loci instead of one, located at different sites and strands of the hmtDNA. In just a few cases these two loci are differentiated by a “1” or “2” added to their qualifiers.

## Abbreviations

CDS: Coding DNA sequence
hmtDNA: Human mitochondrial DNA
MSA: Multiple sequence alignment
PRD: Padded-Recursive-DCM3 decomposition
RefSeq: Reference sequence
rRNA: Ribosomal RNA
tRNA: Transfer RNA

## References

[1] Bader DA, Roshan U, Stamatakis A (2006). Computational Grand Challenges in Assembling the Tree of Life: Problems and Solutions. Adv Comput.

[2] Yang Z, Rannala B (2012). Molecular phylogenetics: principles and practice. Nat Rev Genet.

[3] Grada A, Weinbrecht K. Next-Generation Sequencing: Methodology and Application. J Investig Dermatol. 2013:133(e11). doi:10.1038/jid.2013.248. 10.1038/jid.2013.248 23856935

[4] Edwards AWF, Cavalli-Sforza LL, Heywood VH. Phenetic and phylogenetic classification. Systematic Association Publication No. 6. 1964:67–76.

[5] Cock PJA, Antao T, Chang JT, Chapman BA, Cox CJ, Dalke A, Friedberg I, Hamelryck T, Kauff F, Wilczynski B, de Hoon MJL (2009). Biopython: freely available python tools for computational molecular biology and bioinformatics. Bioinformatics.

[6] Benson DA, Karsch-Mizrachi I, Lipman DJ, Ostell J, Sayers E. W (2010). GenBank. Nucleic Acids Res.

[7] Nelesen S, Liu K, Wang LSK, Linder CR, Warnow T (2012). DACTAL: divide-and-conquer trees (almost) without alignments. Bioinformatics.

[8] Álvarez J, Blanco R, Mayordomo E. Workflows with model selection: A multilocus approach to phylogenetic analysis. In: 5th International Conference on Practical Applications of Computational Biology & Bioinformatics (PACBB 2011). Advances in Intelligent and Soft Computing. Springer Berlin Heidelberg: 2011. p. 39–47.

[9] Álvarez-Jarreta J, de Miguel Casado G, Mayordomo E. PhyloFlow: A Fully Customizable and Automatic Workflow for Phylogenetic Reconstruction. In: IEEE International Conference on Bioinformatics and Biomedicie (BIBM). IEEE: 2014. p. 1–7, doi:10.1109/BIBM.2014.6999303.

[10] Katoh K, Misawa K, Kuma K, Miyata T (2002). MAFFT: a novel method for rapid multiple sequence alignment based on fast Fourier transform. Nucleic Acids Res.

[11] Sievers F, Wilm A, Dineen D, Gibson T, Karplus K, Li W, Lopez R, McWilliam H, Remmert M, Söding J, Thompson J, Higgins D (2011). Fast, scalable generation of high-quality protein multiple sequence alignments using clustal omega. Mol Syst Biol.

[12] Edgar RC (2004). Muscle: multiple sequence alignment with high accuracy and high throughput. Nucleic Acids Res.

[13] Price MN, Dehal PS, Arkin AP (2009). FastTree: Computing Large Minimum-Evolution Trees with Profiles instead of a Distance Matrix. Mol Biol Evol.

[14] Stamatakis A (2006). RAxML-VI-HPC: maximum likelihood-based phylogenetic analyses with thousands of taxa and mixed models. Bioinformatics.

[15] Felsenstein J. Phylogeny inference package (PHYLIP). 2006. http://evolution.genetics.washington.edu/phylip.html.

[16] Löytynoja A, Goldman N (2008). Phylogeny-aware gap placement prevents errors in sequence alignment and evolutionary analysis. Science.

[17] Guindon S, Gascuel O (2003). A simple, fast, and accurate algorithm to estimate large phylogenies by maximum likelihood. Syst Biol.

[18] Swenson MS, Suri R, Linder CR, Warnow T (2012). SuperFine: fast and accurate supertree estimation. Syst Biol.

[19] Merino-Casallo F, Álvarez-Jarreta J, Mayordomo E. Conservation in mitochondrial DNA: parallelized estimation and alignment influence. In: 2015 IEEE International Conference on Bioinformatics and Biomedicine (BIBM 2015). IEEE: 2015. p. 1434–40, doi:10.1109/BIBM.2015.7359887.

